# Modulating Astrocyte Transition after Stroke to Promote Brain Rescue and Functional Recovery: Emerging Targets Include Rho Kinase

**DOI:** 10.3390/ijms17030288

**Published:** 2016-02-26

**Authors:** Hima Charika S. Abeysinghe, Ellie L. Phillips, Heung Chin-Cheng, Philip M. Beart, Carli L. Roulston

**Affiliations:** 1 Neurotrauma Research, Department of Medicine, St Vincent’s Campus, University of Melbourne, Parkville, VIC 3065, Australia; h.abeysinghe@student.unimelb.edu.au; 2 Department of Surgery, St Vincent’s Campus, University of Melbourne, Parkville, VIC 3065, Australia; 3 Department of Biochemistry and Molecular Biology, Bio21 Insitute, University of Melbourne, Parkville, VIC 3010, Australia; ellie_phillips_1@hotmail.com (E.L.P.); heung@unimelb.edu.au (H.C.-C.); 4 The Florey Institute of Neuroscience and Mental Health, Melbourne Brain Centre, Parkville, VIC 3010, Australia; phil.beart@florey.edu.au

**Keywords:** astrogliosis, glial scar, neurovascular unit, regeneration, connectivity, Rho-kinase inhibition

## Abstract

Stroke is a common and serious condition, with few therapies. Whilst previous focus has been directed towards biochemical events within neurons, none have successfully prevented the progression of injury that occurs in the acute phase. New targeted treatments that promote recovery after stroke might be a better strategy and are desperately needed for the majority of stroke survivors. Cells comprising the neurovascular unit, including blood vessels and astrocytes, present an alternative target for supporting brain rescue and recovery in the late phase of stroke, since alteration in the unit also occurs in regions outside of the lesion. One of the major changes in the unit involves extensive morphological transition of astrocytes resulting in altered energy metabolism, decreased glutamate reuptake and recycling, and retraction of astrocyte end feed from both blood vessels and neurons. Whilst globally inhibiting transitional change in astrocytes after stroke is reported to result in further damage and functional loss, we discuss the available evidence to suggest that the transitional activation of astrocytes after stroke can be modulated for improved outcomes. In particular, we review the role of Rho-kinase (ROCK) in reactive gliosis and show that inhibiting ROCK after stroke results in reduced scar formation and improved functional recovery.

## 1. Introduction

Cerebrovascular disease, including ischemic or hemorrhagic stroke, is still the second most common cause of death and disability worldwide. Ischemic strokes are the most frequent, and, for this reason, current treatment focus is recanalization using thrombolytic therapy despite its limited therapeutic window [1]. Decades of research have also focused on discovering and developing neuroprotective agents that may intervene in the biochemical cascades activated during cerebral ischemia. However, with over 200 neuroprotective compounds showing success in preclinical animal models, none have achieved successful translation to clinical practice [2]. Most patients end up with an expanding lesion and functional loss. This relative paucity of curative approaches for stroke has led to widespread evaluation of alternative treatment modalities. In particular, alternative cellular targets outside the neuron—including phagocytic cells, endothelial cells, and astrocytes—present new strategies for preventing the spread of damage. Brain rescue strategies to support surviving neurons in the days after stroke are also under current investigation. In particular, the cells comprising the neurovascular unit might be collectively targeted to retain connections between blood vessels, astrocytes and neurons, rather than targeting individual biochemical cascades within neurons themselves [3]. The neurovascular unit also presents an exciting target for promoting functional recovery, since restoration of nerve transmission after stroke relies heavily on co-ordinated events within the unit. Structural damage to blood vessels and over-activation of astrocytes after stroke, however, results in disassembly of the unit in both damaged and surviving nerve pathways. In particular, nerves on the edge of the damage can be rendered dormant if the neurovascular unit is compromised in this territory. This review will focus on the events associated with changes in the neurovascular unit during and after stroke, with a particular focus on the role of astrocyte transition in the peri-vascular territory and its effects on both lesion and deficit progression, and importantly, functional recovery.

## 2. The Expanding Core

An ischemic attack results in two distinguishable areas: the infarcted core, which is the region supplied by the occluded vessel; and the penumbra, which is the area between the lethally damaged core and normally perfused territory, which receives some collateral blood flow from unaffected vessels [4]. A dramatic reduction in cerebral blood flow (CBF) occurs in the ischemic core leading to depletion of cellular energy supply, changes in ion homeostasis, metabolic processes, and subsequent breakdown of cellular integrity resulting in necrotic cell death [5]. Tissue within the ischemic core is often irreversibly damaged even if blood flow is re-established. The ischemic penumbra, however, can be defined by a moderate reduction in CBF where collateral blood vessels provide cells with limited metabolic nutrients to temporarily maintain homeostasis during the initial stages of ischemia [6]. Despite this, interruption of cellular homeostasis in the penumbral region leads to further cell death where penumbral tissue will be recruited into the infarcted core with a step-by-step spread of damage if reperfusion is delayed [7]. Due to this delayed rate of neuronal loss within the penumbra, the prime goal for therapeutic intervention in the past has been to target salvage of neurons within the penumbra and prevent expansion of the lesion in the h to days post-stroke. However, so many biochemical cascades within neurons are activated synergistically once ion homeostasis is compromised, targeting individual pathways has proved futile since there is not just one single pathway involved in programmed cell death. More importantly however is the influence of age on the expanding lesion, which is often overlooked in pre-clinical studies and requires new considerations, since aging is associated with accelerated infarct development and poor prognosis for full recovery [8]. Given that the majority of stroke patients are above the age of 65, and studies in aged rats show that infarcts progress more quickly, new strategies beyond simply targeting the neuron are desperately required. An approach that targets the key factors that lead to accelerated lesion development in the elderly brain requires urgent investigation.

## 3. The Neurovascular Unit

**Figure 1:**
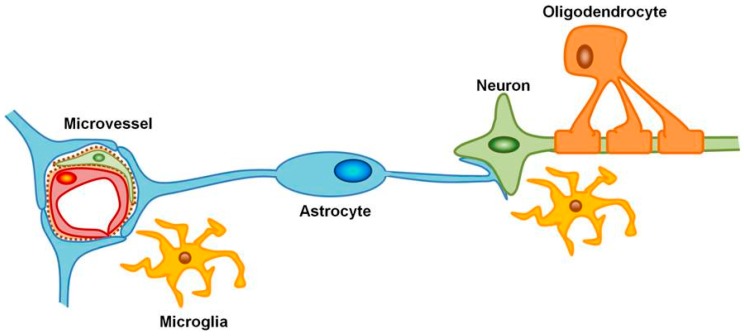
Schematic diagram of the neurovascular unit. The inter-relationship of microvessels and their dependent neurons via astrocytes and surrounding cells including microglia and oligodendrocytes where injury affects the function of the entire unit. Microvessels consisting of pericytes attached to the abluminal surface of the endothelial cells are surrounded by basement membrane and encompassed by astrocyte end-feet.

The study of blood vessels in cerebrovascular diseases has expanded from sole consideration of endothelial cells to include other cells within the practical framework described as the Neurovascular Unit (NVU) [9]. The National Institute of Neurological Disorders and Stroke [10] progress review group identified the NVU as a conceptual model that emphasizes the dynamic interactions between neurons, astrocytes, smooth muscle cells, endothelial cells, pericytes, basement membranes, extracellular matrices and supporting cells (microglia and oligodendroglia) necessary for normal function (Figure 1) [9,11,12,13]. Neurotransmission requires combined efforts within the NVU where astrocytes play a key role in energy storage and transfer, neurotransmitter reuptake and recycling, and maintenance of ion homeostasis. In this respect, astrocytes can be seen as an important link between the cerebral vasculature and neurons, with each neuron often receiving support from up to 50 astrocytes [14]. Following stroke, cells within the NVU undergo considerable change that compromises neuronal survival, nerve transmission and later remodeling events [15]. 

## 4. Changes to the Neurovascular Unit in Stroke

### 4.1. Breakdown of the Blood Brain Barrier

The central nervous system is considered to be immune privileged and is separated from the changeable milieu of blood circulation by the blood brain barrier (BBB) to allow conductivity of complex neural signaling without external interferences [16]. The blood-cerebrospinal fluid barrier established by the choroid plexus epithelial cells works in conjunction with the BBB to create a tight seal preventing passage of blood-borne molecules into the microenvironment of the brain [17]. The BBB comprises of a complex system of highly specialized and unique endothelial cells, their underlying basement membranes where a large number of pericytes reside [18], peri-vascular antigen-presenting cells, ensheathing astrocytic end feet [19] and the associated parenchymal basement membrane [17]. Specific interactions between brain endothelium and astrocytes of the NVU appear to be involved in regulating the BBB under physiological and pathological conditions [19]. Under normal conditions, the tight junction connection with astrocytes only allows the diffusion of very small water-soluble compounds via a par-cellular aqueous pathway, while the lipid membranes of the endothelium comprised of large surface areas offer effective diffusion of lipid-soluble compounds via a trans-cellular lipophilic pathway [20]. 

Cerebral ischemia compromises the BBB integrity for days to even weeks and leads to permeability of previously excluded blood-borne molecules and inflammatory cells [17,21,22]. A cascade of microvascular events contributes to the breakdown of the BBB including fibrin accumulation, transmigration of leukocytes, generation of degrading enzymes, basal laminae breakdown with loss of astrocyte and endothelial cell contacts which causes vasogenic oedema and potential hemorrhagic transformation [17,21,23]. The volumetric effect of oedema formation causes local compression of microcirculation leading to further perfusion deficits, a rise in intracranial pressure, and dislocation of parts of the brain [24]. Indeed, therapeutic strategies that target oedema show potential in animal models but as yet have not translated to clinical practice [25,26]. This may be, in part, due to the failure of these strategies to address the neurovascular unit as a whole, targeting instead vascular cells for restoring the BBB and thus ignoring the critical role of astrocytes in maintaining vascular integrity and BBB stability. 

### 4.2. Vascular Remodeling of Stroke

In response to cerebral vascular compromise after stroke, the brain attempts to restore the BBB through vascular remodeling. Sprouting of new endothelial cells from pre-existing vessels is a process referred to as angiogenesis [27] and includes intussusceptive angiogenesis where one vessels splits in two [28]. Endothelial cell sprouting occurs through migration and mitosis and results in new vessels composed of endothelial cells derived from parent vessels, while intussusceptive angiogenesis occurs by multiplication of existing vessels through lumen splitting. It has been suggested that intussusceptive angiogenesis plays a role in early capillarization, network remodeling as well as large vessel formation. Increased metabolic demand has also been suggested to switch sprouting angiogenesis to intussusceptive angiogenesis for growth of new capillary networks [29]. 

Over 20 endogenous regulators of angiogenesis have been described such as growth factors, matrix metalloproteinases, cytokines and integrins [30,31]. Important roles in the development, induction of endothelial cell division, and differentiation of vasculature occurs through many growth factors including vascular endothelial growth factor (VEGF), VEGF receptor 2, platelet-derived endothelial cell growth factor, fibroblast growth factors (FGFa and FGFb), epidermal growth factor (EGF), angiogenin, and ephrin receptors and ligands [31,32,33]. Transforming growth factor-β and transforming growth factor-α inhibit endothelial cell proliferation but can induce three-dimensional tube formation and other aspects of angiogenesis [31]. Angiopoietins 1 and 2 and their tyrosine kinase receptor Tie2 are involved in maturation, stabilisation, and remodeling of vessels [33]. These regulation factors represent potential mechanisms and molecular targets for angiogenic therapies after brain injury [34,35]. 

Angiogenesis following ischemic brain injury has been demonstrated in experimental models and in humans [36,37,38]. During the early stages of angiogenesis, newly formed blood vessels are permeable and become less leaky as they mature [32,38,39]. Up-regulated expression of VEGF, VEGFR2, angiopoietins, and Tie2 are observed in rodent ischemic brain tissue up to 28 days post-stroke [38,40,41]. Indeed, stroke patients are also observed to have high serum concentrations of VEGF that peaked at 7 days and remained elevated to 14 days [42]. Angiogenesis is essential for ischemic brain repair as it can improve blood flow perfusion to ischemic and surrounding tissues, which then stimulates metabolism and in turn may improve functional outcome following stroke [37]. Indeed a significant correlation between angiogenesis and survival times is reported after stroke in patients, with higher density of blood vessels correlating to longer survival times in comparison to patients with low vascular density [36,42,43]. The degree of angiogenic response after stroke is governed by the initial insult [44] but is also highly influenced by age. Angiogenesis in aged rats has been reported to be diminished due to the persistent upregulation of inflammatory genes and vigorous expression of genes required for the buildup of the fibrotic scar [45]. These factors combined slow down the rate of vascular recovery in the aged brain and directly influence poor functional outcomes [45]. Hence, restorative therapies that enhance angiogenesis have also been associated with improved functional outcomes and suggest, in addition to neurogenesis, that angiogenesis should be further explored as a treatment modality [46,47,48,49]. However, the benefits of angiogenesis can be negatively affected by premorbid disease, as observed in diabetes and/or hypertension and atherosclerosis and this should also be considered when investigating treatment options [33,49]. Additionally, if new blood vessels are not recoupled with trophic astrocyte end feet, the BBB remains leaky.

### 4.3. Astrogliosis

As a dominant cell population in the brain, astrocytes participate in key signaling events associated with neurotransmission including energy transfer, anti-oxidant activity, neurotransmitter up-take and recycling, trophic factor synthesis, ion homeostasis, and neurovascular coupling [50,51,52]. Astrocytes also translate neuronal activity to cerebral vessels via arachidonic acid metabolites and nitric oxide to induce changes in cerebral vascular tone [53]. This information is mediated by the astrocyte end feet via opening of Ca^2+^activated K^+^ channels and the release of K^+^ onto smooth muscle cells to induce vasodilation to support neurotransmission [3]. An intense relationship between astrocytes and neurons, astrocytes and blood vessels is therefore required for normal functioning of the neurovascular unit. 

Astrocytes are recognized as highly plastic cells that exhibit various morphological and biochemical changes triggered by physiological and pathological events that alter their environmental milieu [51,52,53]. Astrocytes can change their physical and molecular phenotype and exist in pro-survival “cytotrophic” and destructive “cytotoxic” distinctions [51,52,53]. Numerous astrocytic mechanisms have evolved to protect neurons from the initial ischaemic insult, including glutamate uptake by astrocytes to prevent excitotoxic accumulation [54]. Metabolite transfer from astrocytes to neurons also provides energy to neurons in the initial phase of stroke, where astrocyte glycogen stores in the early h of stroke serve as a carbon source and cells undergo anaerobic metabolism to support penumbral neurons [54,55]. Further astrocytic support is provided through early detoxification of free radicals as astrocytes contain higher intracellular concentrations of antioxidants than oligodendrocytes and neurons, which contribute to their robust resistance to oxidative stress [56,57]. Reactive astrocytes are known to up-regulate erythropoietin after ischemia, a paracrine messenger within the brain that can prevent apoptosis or excitotoxic stress [57,58,59]. Additionally, astrocytes are now also identified as being important in vascular and neuronal regeneration after stroke through secretion of several known cytotrophic factors important for promoting angiogenesis, directing neural precursor migration, and glial plasticity [60,61,62,63]. Of relevance, Hiryama, *et al.* investigated the role of astrocytes in ischemic tolerance in the brain induced by preconditioning. Their results revealed that pre-conditioning significantly increases the expression of the ATP-gated cation channel P2X7 receptor in astrocytes. The upregulated receptor in turn facilitates development of cytotrophic phenotypes such as expression of hexokinase 2, monocarboxylate transporter 4 and erythropoietin genes [64].

**Figure 2:**
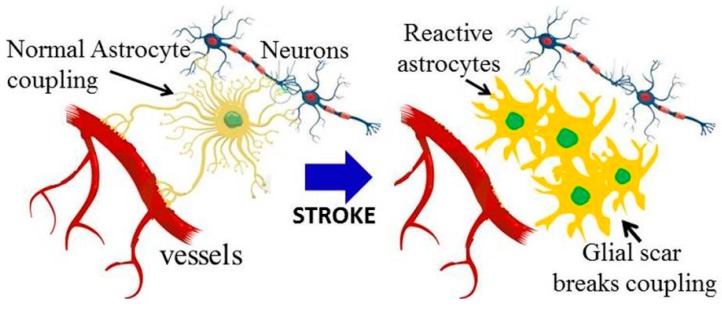
Over activation of astrocytes adjacent to the stroke lesion breaks neurovascular coupling in structurally intact nerves: Schematic diagram of healthy astroctyes with end feet coupling to blood vessels and neurons (long black arrow); following stroke (blue arrow) reactive astrocytes retract their end feet connections to break coupling (short black arrow) to form the glial scar. Targeting astrocytes to reduce the glial scar whilst retaining trophic astrocyte support is a new target for brain rescue.

In contrast to their potential support role, astrocytes actively participate in the demise of brain tissue after stroke if they become over-activated. These over-activated astrocytes (referred to as reactive astrocytes), are characterized by elevated expression of glial fibrillary acidic proteins (GFAPs) [65], and their proliferation is maintained by the Notch1-Stat3-endothelin receptor type B signaling pathway [66]. Astrocyte over-activation results in glutamate accumulation in the synaptic cleft through reversal of volume sensitive ion channels, and down regulation of glutamate transporters, to exacerbate excitotoxicity [54,67]. Reactive astrocytes secrete reactive oxygen species, pro-inflammatory cytokines and interleukins, matrix metalloproteinases, as well as contribute to BBB disruption and facilitate oedema through aquaporin-4 channels abundantly expressed in astrocytic endfeet at the endothelial interface [55,62,68]. Reactive astrocytes amplify ischemic injury through retraction of their end feet from neurons and cerebral blood vessels resulting in the opening of gap junctions. Astrocytes also provide a conduit for the propagation of pro-apoptotic signals such as nitric oxide, TNFα and matrix metalloproteinases between neurons [55]. Collectively, the role of astrocytes after stroke constitutes a finely gradated continuum of morphological changes from reversible pro-survival alterations to long-lasting scar formation around the lesion, a process referred to as reactive gliosis [55,65,66] (Figure 2). Therefore, it is an oversimplification to assign sole protective or destructive functions to astrocytes, and the intricacy of these mechanisms that collectively influence the brain microenvironment after ischemia is highlighted instead.

In an effort to promote recovery after stroke research was initially directed towards globally inhibiting the actions of astrocytes. This adversely affected initial injury and was later abandoned as a therapeutic possibility. Over time, studies revealed that whilst early responses to astrocytes may be necessary, reactivity is governed by negative environmental cues that ultimately lead to a state of over activation. In particular, age has a significant effect on astrocyte activation where reactive gliosis and the premature development of fibrotic scar tissue is reported to be amplified in older subjects, which directly correlates to stagnation in recovery [69]. Important new insights into signaling events within astrocytes in the h after stroke now facilitates a better understanding of how these cells might be specifically targeted to retain their initial functional support. Between six and 24 h after stroke glycogen breakdown in astrocytes becomes impaired [70] resulting in less ATP availability for both neurons and astrocytes, with reduced neurotransmitter re-uptake and recycling from the synapse. This inability to access the glucose reservoir within the brain impairs the role of astrocytes in supporting neural metabolism for neurotransmission. Loss in ATP may also drive changes within astrocytes to initiate reactive morphological transition. Astrocyte shape, movement and cell division is highly governed by the actin cytoskeleton and alterations in gene signaling during stroke drives changes in Notch signaling and Rho GTPases (Rho, Rac, Cdc42) that regulate proliferation and movement [66,71,72]. These changes within the cytoskeleton further impair glutamate re-uptake and turnover through down-regulation of glutamate transporters, EAAT1 and 2 [73,74,75,76,77]. In particular, the glutamate transporter EAAT2 is considered a specific marker for astrocytes with genomic interactions detected with GFAP [78]. Increased expression of GFAP and Rho GTPases, results in cytoskeletal rearrangement and retraction of astrocytic end feet connections from both blood vessels and neurons. Therefore, specific changes within astrocyte signaling pathways may be intimately involved in the varying degrees of reactive astrocyte transition resulting in breakdown of the neurovascular unit.

## 5. The Glial Scar

**Figure 3:**
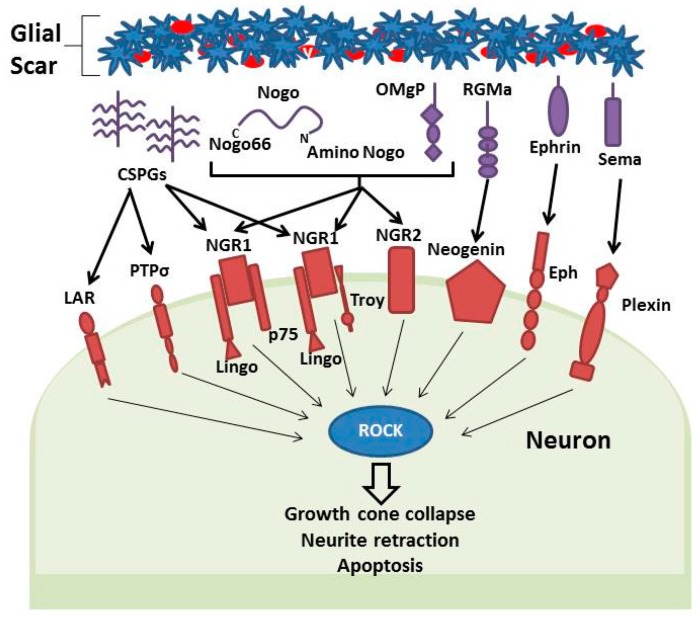
Simple schematic representation of Gliotic scar mediated Rho-kinase (ROCK, blue circle) activation in neurons. Inhibitory molecules released by the glial scar include CSPGs, Nogo (Nogo66 and Amino Nogo), OMgP, RGM, Ephrin and semaphorins. Activation of receptors present on axon membranes (red shapes) signal change in Rho-ROCK activity within neurons resulting in growth cone collapse, neurite retraction and apoptosis. Black arrows reference signaling events from the glial scar that activate receptors on neurons that trigger internal signaling events (Grey arrows) that activate ROCK.

As discussed above, activation of astrocytes following brain injury involves complex chemical and structural modifications that culminate in long-lasting scar formation [52]. Astrocytes within the glial scar appear densely arborized and display a high degree of overlapping processes that are intensely stained for GFAP, which is significantly upregulated with changes in cytoskeletal rearrangement. In addition to activation of pre-existing brain astrocytes, new GFAP positive radial glial cells are also generated in the neurogenic niche of the subventricular zone after stroke, which by three days can be seen migrating towards the intensifying glial scar [44,79]; between 7 and 14 days, large, densely arborized nestin positive astrocytes can be observed around the infarct border [38,44,79], which by 28 days poses a major physical and chemical barrier to the stroke affected brain [38,55,65,79]. Whilst it is important to seal the wound after stroke, the glial scar also spreads into regions of the brain where there are often surviving neurons [38,44]. Whilst many deficits incurred after a stroke are directly associated with neuronal loss within the lesion core, advances in functional Magnetic Resonance Imaging based tractography now show us that loss of electrical activity in otherwise structurally intact pathways also accounts for functional loss [80]. Overactive astrocytes within the glial scar not only represent a physical barrier to neurons trying to re-form connections but are also the source of many inhibitory molecules that prevent axon regrowth [81,82,83] (Figure 3). These inhibitory molecules include chondroitin sulphate proteoglycans (CSPGs) [84,85], ephrins, semaphorins and slit proteins [86,87], which are upregulated by astrocytes following injury. The glial scar also incorporates myelin-associated inhibitory molecules such as Nogo, oligodendrocyte-myelin glycoprotein (OMgp) [88,89] and repulsive guidance molecules (RGM) [90], which are released by oligodendrocytes upon injury. Cultured neurons grown on medium collected from reactive astrocytes show reduced axonal outgrowth compared to neurons grown on control medium [84], due to the inhibitory molecules released by reactive astrocytes. Thus, in addition to changes in chemical metabolism in surviving nerve pathways, changes in astrocyte transition and glial scar formation are also highly inhibitory towards axon regrowth in damaged pathways, thereby preventing neuronal connections from re-forming and hindering functional recovery. Targeting transitional activation of astrocytes after stroke might therefore be an alternative strategy for restoring functional activity in both surviving neurons and damaged nerve pathways, where the trophic effects of activated astrocytes are retained without over activation.

## 6. Modulating Astroctye Transition after Stroke

Investigation into inhibiting the response to astrocytes after stroke resulted in negative effects to both lesion development and functional outcomes. This was clearly demonstrated with transgenic mice lacking proteins known to be involved in astrocyte activation. Following ischemia, knock-out mice lacking the astrocytic intermediate fibres upregulated after stroke (GFAP and Vimentin) showed reduced glial scarring but an increase in infarct size and reduced functional recovery [91,92]. Likewise, conditional deletion of the transcription factor STAT3 in astrocytes following spinal cord injury resulted in reduced glial scarring which was associated with greater damage and increased inflammation [93,94]. It therefore appears that reactive astrocytes limit the spread of neurotoxic molecules by acting as a diffusion barrier as a result of morphological changes and the release of ECM components [95]. These above studies suggest that preventing the initial activation of astroctyes (through gene knockout) blocks the protective role of these cells in the initial stage of stroke. However, what if astrocytes could be modulated to allow initial activation whilst preventing over activation? Indeed studies investigating the effect of environment on recovery after stroke show that astroctyes can be temporally modulated through environmental enrichment with correlated benefits to recovery and reduced scar volume [96]. The use of a pharmacological approach to modulate astroctyes also results in improved outcomes. Chronic immunosuppression with Cyclosporine A prior to and after stroke was reported to attenuate the development of deficits in the h to days after stroke with reduced glial scar formation [97]. This occurred in the absence of neuroprotection or change to microglial responses, but trophic astrocyte morphology identified by spindly, elaborate processes were retained. In this study, astrocytes were shown to undergo transition into a reactive morphology but failed to progress to their over-activated state associated with the glial scar. Due to the absence of neuroprotection, it was therefore suggested that trophic astrocyte support without glial scar formation resulted in brain rescue where neurotransmission in regions of the brain that would otherwise succumb to functional loss, due to scar progression, were retained. 

Whether activated astrocytes are beneficial or an impediment is therefore largely due to timing and the extent of reactive astrogliosis. Whilst initial activation limits damage in the early stages of stroke, over-activated astrocytes form a persistent glial scar that disrupts nerve transmission and impedes later recovery [98]. Identifying intracellular signaling pathways that can modulate astrocyte responses more specifically after stroke is therefore an exciting new approach to treating stroke. 

### 6.1. The Rho/ROCK Pathway

**Figure 4:**
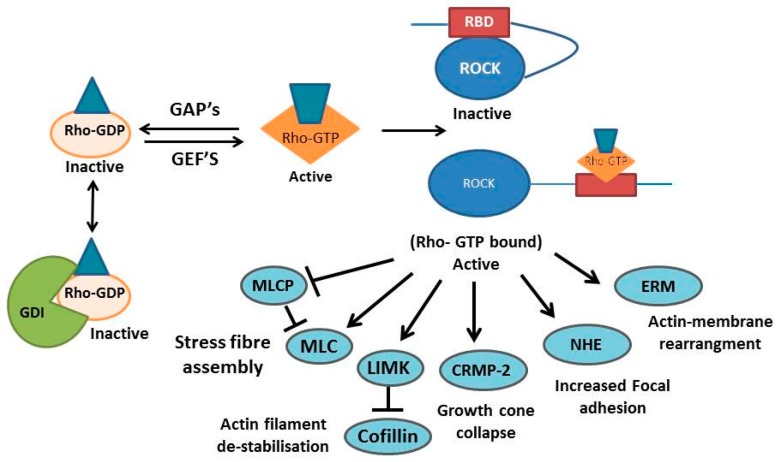
The Rho/ROCK signaling pathway. Rho is kept in the inactive GDP-bound state (yellow/blue complex) by sequestration with Guanine nucleotide Dissociation inhibitors (GDIs; green comples) and the activity of GTPase activating proteins (GAPs). Rho can be activated through Guanine Exchange Factors (GEFs), enabling the exchange of GDP for GTP. Rho-GTP (orange/blue complex) can then activate ROCK (blue/red complex) by binding to the Rho binding domain. Active ROCK can then phosphorylate multiple downstream effectors eliciting changes in actin membrane stabilisation, growth cone collapse and increased cell adhesion, which in astrocytes results in glial scar formation. Arrows indicate activation events, whereas blunted lines indicate inhibitory effects.

Chronic immunosuppression is not a favorable pharmacological approach to treating stroke. Therefore alternative strategies must be explored. One potential strategy might be to target intracellular pathways involved in over-activation of astrocytes. One such pathway is the Rho/ROCK pathway, the inhibition of which could be used to disrupt glial scarring while maintaining the initial beneficial effects of activated astrocytes. Rho and its downstream effector, the Rho associated coiled coil protein kinase (ROCK), form an important pathway that regulates motility and cytoskeletal structure. Rho, a family of the small GTPases can switch between inactive GDP-bound and active GTP-bound states. This switching is controlled by Guanine Exchange Factors (GEFs) and GTPase Activating Proteins (GAPs). GEFs enhance activation by aiding the substitution of GDP for GTP. Conversely GAPs inactivate Rho by increasing the hydrolysis of GTP into GDP. In addition, Guanine nucleotide Dissociation Inhibitors (GDIs) bind and sequester inactive Rho in the cytoplasm, preventing localization to the membrane, the site of Rho activity [99]. Active GTP bound Rho can bind and activate its downstream effector ROCK (Figure 4). 

ROCK has multiple downstream effectors, which it phosphorylates to elicit changes to cell morphology and migration. These substrates are involved in multiple processes including: (1) Cytoskeletal rearrangement via stress fiber formation and actin polymer stablization. Stress fiber assembly is coordinated through myosin light chain (MLC) phosphorylation as well as phosphorylation and subsequent inhibition of myosin light chain phosphatase (MLCP) [100]. Actin polymer stabilized by phosphorylation of LIM kinase which inhibits co-filin; (2) Growth cone collapse via Collapsin Response Mediator Protein (CRMP-2) activation; (3) Focal adhesion formation via Sodium–hydrogen exchanger (NHE) activation [99]; (4) Association between actin filaments and membranes through ERM phosphorylation [101]; and (5) Apoptosis [102]. 

### 6.2. The Rho/ROCK Pathway in Stroke 

Many studies have reported the up-regulation of the Rho/ROCK pathway after stroke. Increased Rho and ROCK expression have been observed in neurons and astrocytes of the ischemic hemisphere of rodents and human within h of stroke [103] resulting in increased ROCK activity [104]. In astrocytes, increased ROCK activity is implicated in reactive astrogliosis and glial scar formation. However, this effect was initially debated due to problems associated with the use of astrocyte cultures. Cultured astrocytes have very different morphologies to healthy astrocytes of the brain. Rather than the star-like shape with many fine projections observed in native astrocytes, cultured astrocytes adopt a flat polygonal morphology, which is actually more akin to reactive astrocytes. Indeed, the transcriptome of cultured astrocytes is now known to be closer to that of reactive astrocytes than healthy brain-derived astrocytes [105]. Debate over what constituted a reactive astrocyte initially led to ROCK inhibitors being thought to induce reactivity as cultures treated with ROCK inhibitors adopt a stellate morphology [73,105]. However, using modern techniques such as 3D culturing [76,77] and with greater appreciation of astrocyte heterogeneity, it is now apparent that ROCK inhibition results in reduced astrocyte reactivity [73,74,75,76,77]. Changes in actin dynamics are responsible for the morphological changes associated with reactive astrogliosis [100]. These changes are controlled by Arp2/3 signaling, which lies upstream of the Rho/ROCK pathway. Astrocytic cultures that adopt a reactive morphology following Arp2/3 inhibition have been shown to have a higher proportion of active GTP-bound Rho than inactive GDP-bound Rho. These morphological changes are reversed upon ROCK inhibition [106]. Thus, it would appear the Rho/ROCK pathway is essential for reactive astrogliosis. 

In neurons, ROCK activity is also upregulated in response to multiple inhibitory molecules released by the glial scar following a stroke (see Figure 3). These inhibitory molecules also include CSPGs, myelin-associated inhibitors, ephrins, semaphorins and slit proteins [90,107,108,109] lead to ROCK activation in axons [108,109,110]. ROCK in turn phosphorylates CRMP-2 leading to growth cone collapse and inhibition of axon growth [99,108,110]. Inhibition of the Rho/ROCK pathway has been found to attenuate the growth cone collapse caused by these inhibitors, with greater axon length and sprouting observed in neurons grown on medium containing CSPGs and myelin-associated inhibiters when treated with ROCK inhibitors [107,108,110,111,112]. This enhanced nerve outgrowth is also seen in rats with spinal cord injury that have been treated with ROCK inhibitors, leading to improved functional recovery [108]. ROCK-mediated axon growth inhibition prevents the re-formation of damaged neuronal pathways following a stroke and so prevents effective functional recovery. Therefore, inhibition of ROCK may promote recovery by minimizing the effects of inhibitory molecules released by the glial scar on neurite regeneration and outgrowth. 

### 6.3. Inhibition of the Rho/ROCK Pathway

Fasudil is an isoquinoline sulphonamide derivative that inhibits ROCK activity by binding to its ATP binding site. The isoquinoline of Fasudil acts as a competitive inhibitor by occupying the same site as ATP’s adenosine ring [113,114]. Fasudil is selective for ROCK, but, due to the similar structure of many kinase ATP binding sites, it can also bind to Protein kinase A (PKA) and AMP-activated protein kinase (AMPK) with lower affinity. However, in the body, Fasudil is quickly converted to its metabolite, hydroxyl-Fasudil, which has a much higher specificity for ROCK [114]. Currently, Fasudil has been clinically trialed to treat vasospasm [115] and is under investigation for the treatment of other diseases including amyotrophic lateral sclerosis [116], multiple sclerosis [117] and spinal cord injury [118]. Notably, a placebo-controlled double-blind clinical trial has been conducted using Fasudil to treat stroke [119]. This resulted in significant improvements in both functional and clinical outcomes, albeit with low cohort numbers. 

Multiple mechanisms have been identified through Rho-kinase inhibition. It has been found to increase cerebral blood flow by preventing down regulation of endothelial nitric oxide synthase [120] and to act as a neuroprotectant, reducing neuronal death by inhibiting apoptotic pathways [121]. While these effects are beneficial, like all neuroprotectants they depend on the early administration. However, mechanisms that are also associated with brain recovery have also been proposed. Firstly, fasudil attenuates the inhibition of axon growth associated with CSPGs released from reactive astrocytes [122]. Other Rho/ROCK inhibitors have also been effective at reversing inhibition caused by the inhibitory molecules of the glial scar [107,108,110,111,112]. Furthermore, Fasudil stimulates astrocytes to produce increased levels of Brain derived neurotrophic factor and granulocyte colony stimulating factor and upregulates glutamate transporters (EAATs) [74,77]. G-CSF is also a growth factor associated with increased neurogenesis, so its upregulation could promote neuronal differentiation and subsequent functional recovery [122].

Pretreatment with Fasudil prior to stroke has been found to result in improved functional outcomes, reduced infarct size, less neuronal death and less BBB permeability [120,121,123]. Of course, pretreatment is not a viable option for real stroke patients. Administering Fasudil from between five minutes to 6 h after stroke has also been shown to result in improved functional outcomes, with the same neurological effects as pretreatment [124,125]. Only one study has significantly delayed treatment until after the acute phase of stroke, where Fasudil treatment commenced at three days and still resulted in improved functional recovery [126]. When delivered soon after stroke, Fasudil is believed to be beneficial by increasing vasodilation and reducing apoptosis, as discussed earlier. However, following the acute phase of stroke once damage has matured, delayed treatment with Fasudil is unlikely to contribute to functional improvement through these actions. For this reason, Lemmens *et al.* [126] attributed the effects of delayed treatment with Fasudil to interruption of the axon growth inhibitor EphA4 which is released by the glial scar. Targeting inhibitory actions on the nerve growth cone from the glial scar, Fasudil is therefore reported to support nerve regeneration and recovery. However, the effect on the glial scar itself was not assessed.

### 6.4. Rho/ROCK Inhibition Modulates Reactive Gliosis after Stroke

Despite the recent interest in Fasudil, its effect on astrocytes and the neurovascular unit in an *in vivo* model of stroke have not been widely explored. Using *in vitro* models, Fasudil treatment of cultured astrocytes results in morphologies and gene expression associated with less severe astrogliosis [74]. As discussed above, these included increased expression of glutamate transporters and growth factor BDNF, as well as decrease in Aquporin-4 associated with BBB dysfunction [73]. Combined, these modulations alone would be expected to benefit functional recovery and retain the role of astrocytes within the NVU. 

**Figure 5:**
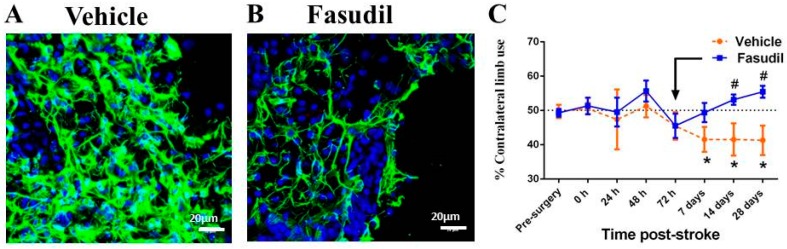
Diffuse astroctyes (GFAP; green, DAPI; blue) within the peri-infarct cortex of vehicle rats at 28 days post stroke (**A**) are attenuated by fasudil (10 mg/kg/i.p./daily) (**B**); Treatment with fasudil commencing three days after stroke significantly improved contralateral forepaw deficit by 14 days (**C**). * *p* < 0.05 *vs.* 0 h scores; # *p* < 0.01 *vs.* vehicle treatment; *n* = 7/group (unpublished data, [128]).

Although not a stroke model, the effect of Fasudil on glial scarring has been investigated on injured retinas *in vivo* [127], where it was demonstrated that Fasudil reduced reactive astrogliosis and glial scarring, which was associated with increased neuronal survival. However, as different injury models are associated with different protein expression patterns for reactive astrocytes, it is important to investigate the effects of Fasudil on astrocytes using a clinically relevant stroke model to properly characterize its effect as a treatment for stroke. To date, no study has reported the effect of Fasudil on transitional changes in astrocytes after stroke *in vivo*. Studies in our laboratory, however, have recently found that Fasudil treatment commencing three days after stroke indeed modulates reactive gliosis to reduce glial scarring in rats, as evidenced by reduced GFAP staining and the adoption of less reactive morphologies up to 28 days (Figure 5B) [128] (unpublished data). Importantly modulation of the astrocyte response resulted in time dependent reversal of functional deficits in the absence of neuroprotection (Figure 5C) [128].

The model of stroke chosen for this study was the endothelin-1 model of conscious stroke in rats based on recent quantification and characterization of multiple remodeling events shown to be similar to that in humans [44]. In this model of middle cerebral artery occlusion, neuronal loss to the dorsal motor cortex is rarely observed, yet clear motor deficits to the contralateral forepaw are detected and persist over time. This is in keeping with MRI studies in humans where functional loss is detected in regions of the brain where there are surviving neurons. We suggest that surviving neurons within the perifarct territory are in a state of inactivity due to breakdown in the NVU and expansion of the glial scar, resulting in functional loss to an otherwise undamaged structure. Treatment with Fasudil potentially targets this area by modulating astrocytes to retain trophic support for brain rescue and neurovascular coupling to prevent loss of neurotransmission in surviving nerve pathways. This of course warrants further extensive investigation to address this hypothesis, but it does offer an alternative strategy for targeting brain rescue and recovery of the stroke-affected brain after stroke.

## 7. Conclusions

Breakdown in the neurovascular unit after stroke involves reactive morphological transition of astrocytes that initially protect the brain against the ischaemic insult but ultimately results in extensive glial scar formation and spread of injury. The glial scar is also a major impediment to the recovery processes through lost neural connectivity in surviving pathways due to breakdown in the neurovascular unit, as well as preventing nerve regeneration through inhibition of axonal growth cone extension. Whilst inhibiting the initial activation of astrocytes after stroke results in bad outcomes, modulating this response might be better approach, where initial responses by astrocytes are preserved, but overall glial scarring reduced. The Rho-kinase pathway is a potential target for modulating this effect. Its inhibitor, Fasudil, has previously been investigated as a potential neuroprotectant following stroke, with positive results. However as ROCK is involved in multiple signaling pathways, it is likely to have an additional mechanism. As such, new evidence suggests that inhibition of ROCK stabilizes astrocytes after stroke by retaining their trophic reactive phenotype without over activation and scar formation, even when treatment is significantly delayed *in vivo*. This approach results in better long term functional outcomes and importantly highlights the need to address different approaches to treating stroke beyond targeting specific events within neurons.
